# Characterizing geometrical distortions in MRI for radiotherapy: Evaluating MR‐SIM protocols and dose accuracy in stereotactic radiosurgery using AAPM TG‐284 criteria

**DOI:** 10.1002/acm2.70199

**Published:** 2025-09-30

**Authors:** Mojtaba Barzegar, Aram Rostami, Abbass Yousef Mkanna, Tarraf Torfeh, Satheesh Prasad Paloor, Ahamed Basith, Souha Aouadi, Rabih Hammoud, Noora Al Hammadi

**Affiliations:** ^1^ Radiation Oncology Department National Center for Cancer Care and Research Doha Qatar; ^2^ Society for Brain Mapping and Therapeutic Los Angles California USA

**Keywords:** dosimetric impact, MR geometric distortion, SRS

## Abstract

**Objective:**

Magnetic resonance imaging (MRI) is crucial for diagnostic imaging and radiotherapy (RT) planning due to its superior soft tissue contrast. However, geometric distortions can affect treatment accuracy. This study evaluates the geometric accuracy of MRI protocols using a 1.5T MR‐Sim scanner for RT and their dosimetric impacts.

**Materials and Methods:**

Geometric distortion was assessed using the CIRS 604‐GS MR Image Distortion Phantom with 2152 control points across various MRI sequences. A central cubic structure and surrounding regions were analyzed, totaling 27 structures. Rigid registration in Eclipse V18.1 aligned MRI images to CT images, with verified accuracy and visual inspection. An evaluation of the overlap and surface distance metrics was performed through MIM Mastro. Geometric distortion was quantified using 3D distortion analysis software, comparing marker coordinates to reference CT scans. Twenty‐seven VMAT full arc plans were done in Eclipse on 27 paired structures, irradiated with 16 Gy in a single fraction at 6 MV energy using the Acuros XB algorithm

**Results:**

All 3D sequences demonstrated mean distortions of 0.4–0.5 mm, with maximum distortions of up to 3.3 mm in the TOF (time of flight) Angiography sequence. In contrast, the T2 TSE 2D sequence showed larger distortions (mean 1.4 mm, max. 9.7 mm). The center structure showed stable dosimetric performance (D_mean_: 1601.6 cGy for CT, 1600.3 cGy for MR). Peripheral regions showed higher variability, with MR_normalized_ D_95%_ values ranging from 83.9% to 88.1%.

**Conclusion:**

Precise MR protocols are essential for accurate tumor delineation and RT planning. While all sequences showed acceptable accuracy, 3D sequences are superior for high‐precision RT.

## 1 INTRODUCTION

Magnetic resonance imaging (MRI) has become an indispensable tool in medical imaging, serving as a cornerstone in both diagnostic imaging and radiotherapy (RT) planning.^1^ Its ability to produce detailed images of soft tissues, compared to computed tomography (CT), is invaluable for detecting, delineating, and accurately characterizing a wide range of pathologies with enhanced precision.^2^

Although MRI can provide detailed information about healthy organs and tumor boundaries, the geometric accuracy of these boundaries can be compromised by distortions due to static magnetic field inhomogeneity, gradient field nonlinearity, tissue magnetic susceptibility, and chemical shift artifacts. RT relies heavily on precise imaging for tumor delineation, as even minor distortions can lead to misdiagnosis or improper treatment of lesions. These images are crucial for ensuring that the prescribed dose is precisely delivered to the targeted lesions.^3^

The extent of these distortions varies depending on the MRI system, the type of sequences, and the specific sequence parameters.^4^ MR‐Sim parameters and specifications differ from those used in diagnostic imaging, being specifically adjusted to minimize geometrical distortions and related artifacts. In advanced, high‐dose treatment modalities such as Gamma Knife or CyberKnife (CK), MRI is increasingly used for its superior ability to delineate structures.^5^

However, reliance on MRI can lead to geometric inaccuracies that may affect dose distribution and compromise treatment safety. This issue becomes particularly critical in advanced RT techniques, such as stereotactic radiosurgery (SRS) for the treatment of AVMs (arteriovenous malformation) and brain metastases, which deliver high doses in just a few sessions.^3^, ^6^, ^7^ Accurate identification of anatomical structures and abnormalities is essential, as any deviation from true anatomy can lead to inaccuracies in dose distribution. Clinically, since no planning margin is typically added to the GTV (gross tumor volume) in SRS, misdelineation can compromise treatment efficacy. Additionally, deviations may increase the risk of harm to surrounding healthy tissues, particularly organs at risk (OARs), where minor inaccuracies can result in unintended dose exposure.^8^, ^9^, ^10^, ^11^, ^12^, ^13^, ^14^

Given these high stakes, maintaining the highest possible geometrical fidelity in MRI is essential for achieving the best clinical outcomes in RT.^5^ This study evaluates the geometrical accuracy of MRI protocols and their dosimetric impact using a dedicated GE MR‐Sim 1.5T scanner in radiation therapy treatment planning. By assessing CK protocols for brain, spine, and AVM imaging, the research aims to determine the performance and adherence to the American Association of Physicists in Medicine (AAPM) Task Group 284 report (AAPM TG‐284) and ACR (American College of Radiology) MRI QC guidelines.^15^, ^16^ The goal is to measure MRI geometrical accuracy, which is crucial for effective patient care in therapeutic settings, and to evaluate the dosimetric impact of MRI distortions on RT treatment planning. By comparing the geometrical accuracy of MRI protocols in different clinical contexts and assessing their dosimetric consequences, this research provides critical insights for optimizing imaging protocols.

## 2 MATERIALS AND METHODS

### 2.1 Phantom and MRI equipment

A resolute CIRS Model 604‐GS Large Field MR Image Distortion Phantom (Sun Nuclear, Virginia, USA) was employed in this study. This liquid‐fillable acrylic phantom has an outer diameter of 356 mm, a length of 300 mm, and a height of 270 mm, featuring a proprietary 3D grid structure of 3 mm diameter rods arranged orthogonally. With 2152 control points per CAD model, it enables comprehensive distortion assessment beyond 2D phantoms.

The phantom material has a mean Hounsfield unit (HU) value of 273 ± 5 and a relative electron density (RED) of 1.19, ensuring accurate dose calculations. It allows for evaluating image distortions arising from B₀ inhomogeneity, gradient nonlinearity, chemical shift, and susceptibility artifacts.

The study employed a wide bore (70 cm) 1.5T GE‐MR SIGNA Artist (GE Healthcare, Milwaukee, Wisconsin, USA) scanner used for RT simulation. For CT image acquisition, we employed a Siemens SOMATOM Definition AS (Siemens Healthcare GmbH, Erlangen, Germany) scanner (64‐slice, dual‐source configuration), which provides high‐resolution imaging essential for accurate target delineation, and stable HU consistency.

### 2.2 Imaging procedure

Prior to scanning, all relevant quality assurance (QA) tests for MRI (AAPM TG‐284)‐ including central frequency of the magnetic field verification and B_0_ inhomogeneity‐ and for CT (AAPM TG‐66) were thoroughly performed and passed to ensure the integrity and accuracy of the process.

#### 2.2.1 MR‐Sim imaging

For 3D QA scans, the phantom's crosshair was meticulously aligned with the isocenter. Each sequence and its parameters listed in Table 1 were individually applied to cover the entire stationary 3D phantom^20^. For all imaging sessions, a flat patient couch overlay was used to position the phantom, replacing the standard curved diagnostic imaging couch. MRI acquisition employed an AIR 20‐channel coil in combination with a 40‐channel surface couch coil. The phantom was aligned using a precise laser system with its crosshairs and positioned at the machine isocenter. All MRI sequences incorporated the manufacturer's 3D gradient nonlinearity correction algorithm to minimize geometric distortions.

**TABLE 1:** MRI sequences and parameters for CyberKnife applications at 1.5T.

MR sequence		TR (ms)	TE (ms)	BW (Hz/Px)	Thickness (mm)	Acquisition matrix size	Acceleration: HyperSense*Phase	Total time
Name	Type							
T1 MPRAGE 3D	SPGR	2438	2.7	220	0.6	224*224	1.0* 2.0	3’50”
T2 FLAIR 3D	SE_IR	5500	89	220	0.6	256*256	1.2* 2.0	4’28”
T2 Cube 3D Brain	SE	2500	100	220	0.6	256*256	1.0* 2.0	3’58”
T2 Cube 3D Spine	SE	2000	90	220	0.5	288*288	2.0* 2.0	4’07”
TOF MRA (AVM)	SPGR	24	2.3	220	0.5	360*360	2.0* 2.0	5’23”
T2 TSE Tra 2D	TSE	7739	97	220	3.0	256*256	1.0* 3.0	5’02”

Abbreviations: Px.BW, pixel bandwidth; SE_IR, Spin Echo Inversion Recovery; SE, Spin Echo; SPGR, Spoiled Gradient Echo; TSE, Turbo Spin Echo; TR, repetition time, TE, echo time.

#### 2.2.2 Geometric correction option

In GE MR scanners, during imaging, the 3D geometrical correction option should be used to minimize the geometrical inaccuracy. This 3D geometric correction option compensates for distortions by mapping coordinates from the distorted image space (*x*_0_, *y*_0_, *z*_0_) to the undistorted space (*x*, *y*, *z*) through re‐sampling using interpolation methods. This correction relies on known static magnetic field (B_0_) and gradient field profiles, with spherical harmonics used to represent distortions caused by magnetic field inhomogeneities and gradient non‐linearities. The spherical harmonic coefficients, derived during shimming and field mapping, are applied by correction algorithms to improve image accuracy. The correction equation (Equation 1) is expressed as:

(1) $$\left[\begin{array}{c} x\\ y\\ z \end{array}\right]=\left[\begin{array}{c} x_{0}\\ y_{0}\\ z_{0} \end{array}\right]+\sum_{l=0}^{L}\sum_{m=-l}^{l}a_{lm}.Y_{l}^{m}\left(\theta ,\emptyset \right)$$

where:

1. *x*, *y*, and *z*, are the coordinates in the undistorted space (target coordinates).
2. *x*_0_, *y*_0_, and *z*_0_ ​ are the coordinates in the distorted space (measured coordinates).
3. $a_{lm}$ are the spherical harmonic coefficients, representing the strength of each harmonic term.
4. $Y_{l}^{m}\left(\theta ,\emptyset \right)$ are the spherical harmonic functions, which depend on the spherical coordinates θ (azimuthal angle) and ϕ (polar angle).
5. l and m are the spherical harmonic degree and order, respectively, and L is the maximum degree considered for the correction.

This transformation adjusts the distorted coordinates (*x*_0_, *y*_0_, *z*_0_) to their corrected positions in the undistorted space, helping to eliminate distortions caused by magnetic field inhomogeneities and gradient non‐linearities.

#### 2.2.3 CT‐Sim imaging

For CT simulations, a flat overlay patient couch top specifically designed for RT was used to minimize positioning uncertainties. The phantom was aligned using an external laser system with crosshairs to ensure precise alignment with the machine isocenter. By following clinical positioning protocols, setup consistency was ensured, and potential uncertainties were minimized across imaging sessions. CT images acquired in helical mode at 140 kVp, 220 mA, 400 mAs, and a slice thickness of 0.6 mm with a 512 × 512 acquisition matrix, served as the gold standard reference.

### 2.3 Image processing, registration, and contouring

**FIGURE 1:**
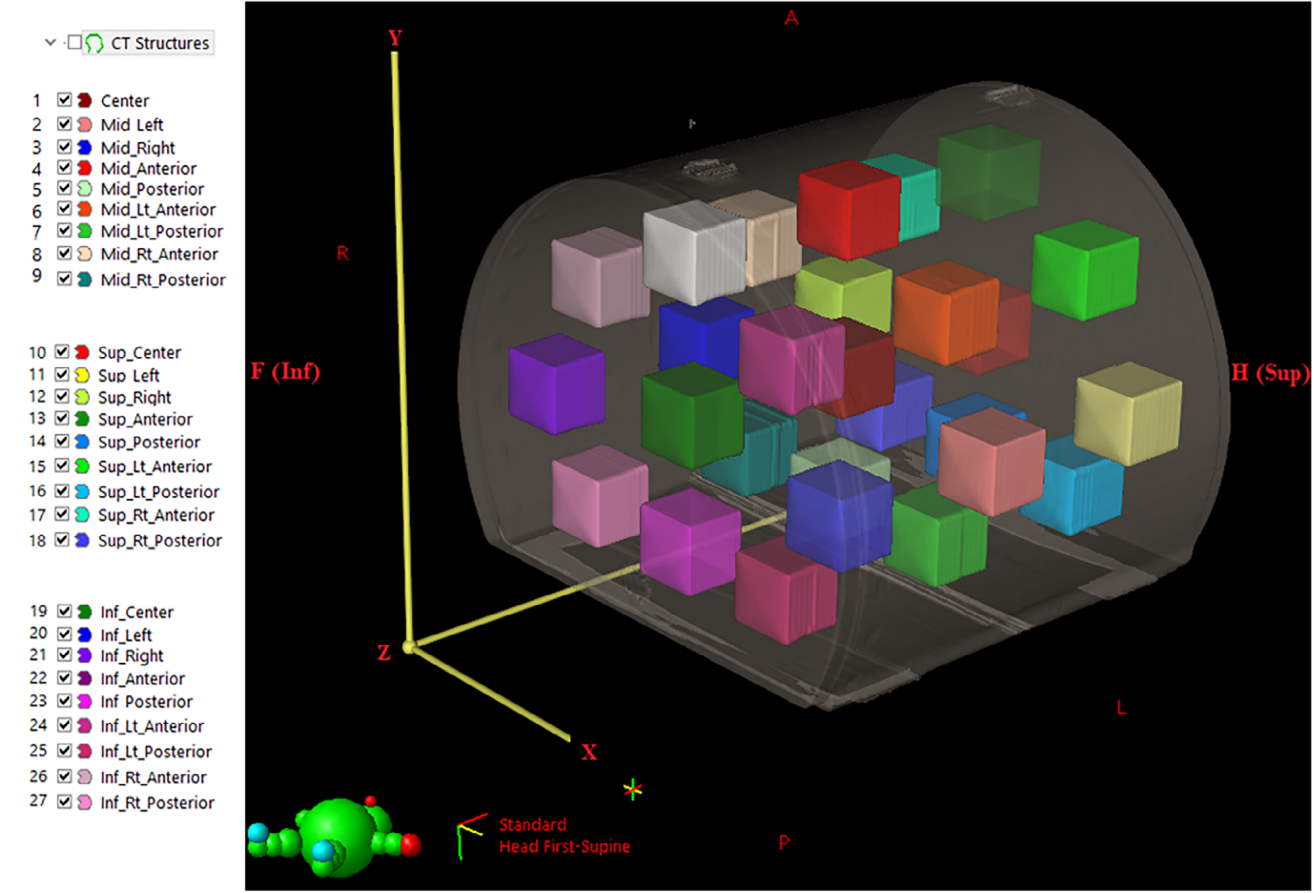
Contoured structures in various positions surrounding the central structures; Note that the center structure is located precisely at the isocenter of the phantom/magnet. The coordinate system follows the radiotherapy convention: X (L–R), Y (A–P), and Z (Sup‐Inf).

To investigate the impact of geometric distortions on dose distribution, three central cubic structures were added at the isocenter (X = 0, Y = 0, Z _=_ 0), Head (Cranial) side (X = 0, Y = 0, Z = −12.24 cm), and Foot (Caudal) side (X = 0, Y = 0, Z = +12.24 cm). Each central structure was surrounded by eight uniformly sized structures (∼100 cm^3^ each) positioned within the phantom periphery across the middle, superior, and inferior sections. This resulted in a total of 27 paired CT/MR structures (Figure 1).

For contouring, each structure was delineated by connecting points along the edges and boundaries. The process involved manually identifying control points on each structure, followed by interpolation to connect the points and reconstruct the 3D volume of the structures. This approach ensured precise delineation of each volume within the phantom, accounting for potential geometric distortions and allowing for accurate evaluation of their impact on dose distribution.

The challenges in merging MRI and CT data primarily arise from patient (phantom) positioning and scan parameters. MRI–CT registration addresses the lack of electron density information in MR‐based RT by spatially aligning MR and CT images. Once co‐registered, the voxel information from both modalities is combined. Most co‐registration techniques treat the imaged volume as a rigid body, and the transformation of coordinates is represented by a linear, spatially invariant function (Equation 2):

(2) $$r_{1}=A.r_{2}+b$$

here, “***r”*** represents the coordinates of corresponding points in the CT (primary dataset: r1) and MRI (secondary dataset:r2) images. The matrix “***A”*** includes rotation, scaling, and plane reflection operations, while vector “***b”*** accounts for translation. Radiation doses are calculated using CT data, which provides attenuation values in HUs that are converted into electron density for tissue‐specific dose calculations.

Rigid image registration was performed using the image registration module in Eclipse V18.1 (Varian Medical Systems Inc., Palo Alto, CA), with MRI images initially aligned to the reference CT images. Image rigid registration can be performed automatically or manually. Manual registration uses fiducial points, typically requiring at least eight points for accuracy, while automated employs automatic algorithms. Landmark selection is critical, ensuring widely spaced points for precise registration.

**FIGURE 2:**
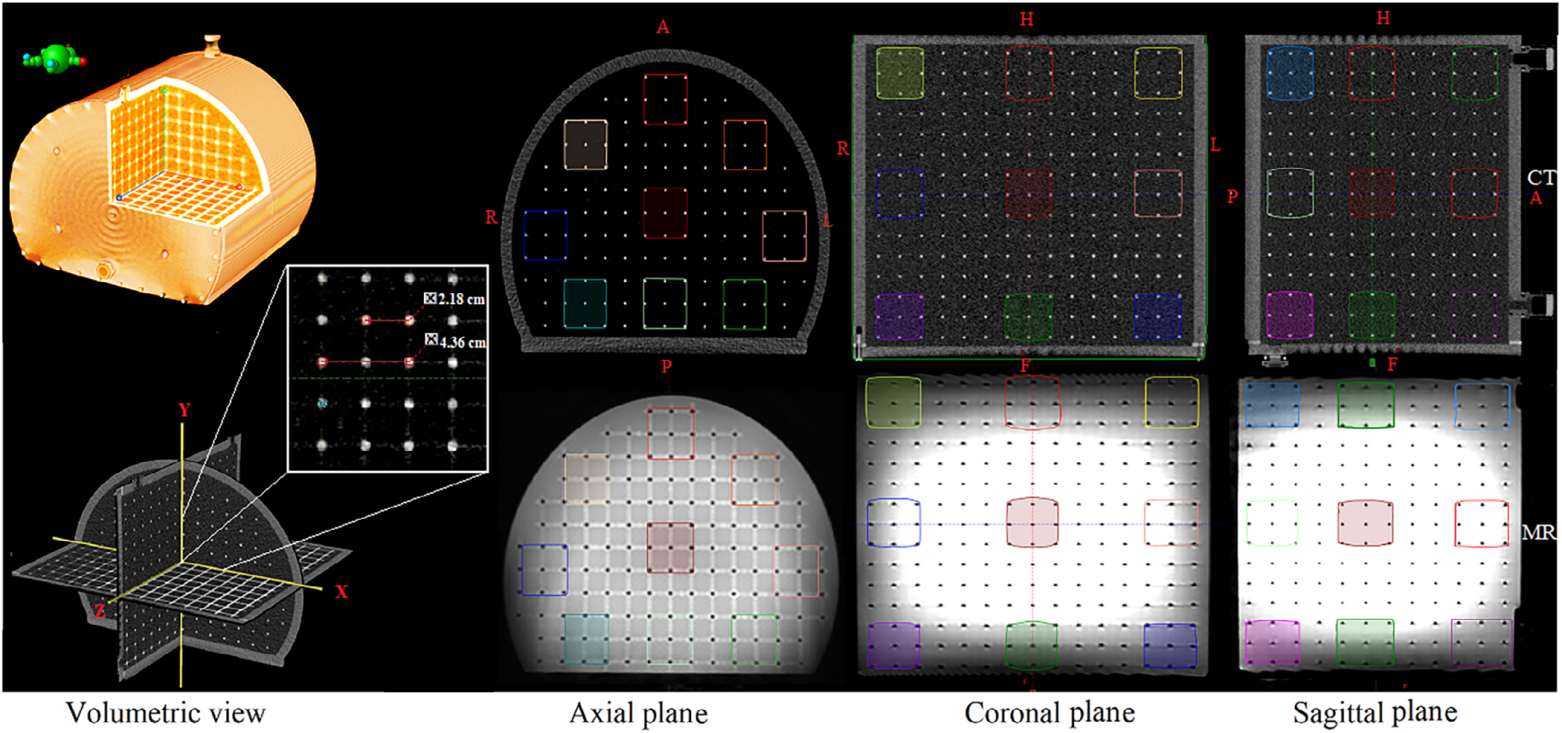
Image scans showing a set of contours for CT (top) and MR (bottom) in three orthogonal planes intersecting at the isocenter for the 3D MR phantom shown on the left.

Accuracy of registration was verified through “target registration error” option in the Eclipse software with point pairs as well as visual inspection according to the recommendations of AAPM TG‐132.^17^ After completing image registration, all contoured structures in MRI datasets were transferred to CT datasets. Following registration, contours were visually verified to confirm correspondence, enabling accurate dose calculation and assesment of geometric distortion effects on dose distribution across the volumes (Figure 2).

In this study, volume contouring accuracy was evaluated through MIM Maestro (MIM Software Inc., Cleveland, OH, USA) using three contour similarity metrics: Dice Similarity Coefficient (DSC), Jaccard Index (J), and Hausdorff Distance (HD). DSC measures the spatial overlap between two contours, ranging from 0 (no overlap) to 1 (perfect overlap). The Jaccard index, a metric like DSC, quantifies the percentage of overlap between two regions relative to their combined area, where values closer to one indicate better agreement in Equations (3) and (4).

(3) $$DSC_{\left(A,B\right)}=\frac{2\left|A\cap B\right|}{\left|A\right|+\left|B\right|}$$

(4) $$J_{\left(A,B\right)}=\frac{\left|A\cap B\right|}{\left|A\cup B\right|}=\frac{\mathrm{DS}C_{\left(A,B\right)}}{2-\mathrm{DS}C_{\left(A,B\right)}}$$

where |A∩B| is the overlap between *A* and *B* and |A∪B| is the union of ***A*** and ***B***. HD measures the maximum distance between corresponding points on two contours, highlighting areas of significant discrepancy across various structures​. In this study, we primarily focused on the Jaccard index because it provides a stricter measure of contour similarity, while including DSC and HD for a comprehensive evaluation.^19^

### 2.4 3D absolute geometrical distortion

**FIGURE 3:**
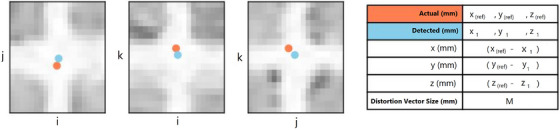
Image display of expected (orange) and measured (blue) control points. The spatial deviation between these points represents the measured distortion within the image volume.

Geometric distortion analysis was conducted using CIRS’ Distortion Check software. This software compares the detected marker coordinates from the 3D MRI scans to their reference positions in the CT space, generating detailed 3D distortion vector fields. To account for global positioning offsets, the software applied a registration step before computing the distortion values. The absolute geometric distortion (GD) corresponding to each control point is given by Equation (5):

(5) $$GD=\sqrt{{\left(x_{1}-x_{ref}\right)}^{2}+{\left(y_{1}-y_{ref}\right)}^{2}+{\left(z_{1}-z_{ref}\right)}^{2}}$$

where (see also Figure 3):

1. $\left(x_{1},y_{1},z_{1}\right)$: Measured point coordinates.
2. $\left(x_{ref},y_{ref},z_{ref}\right)$: Expected (reference) point coordinates.

**FIGURE 4:**
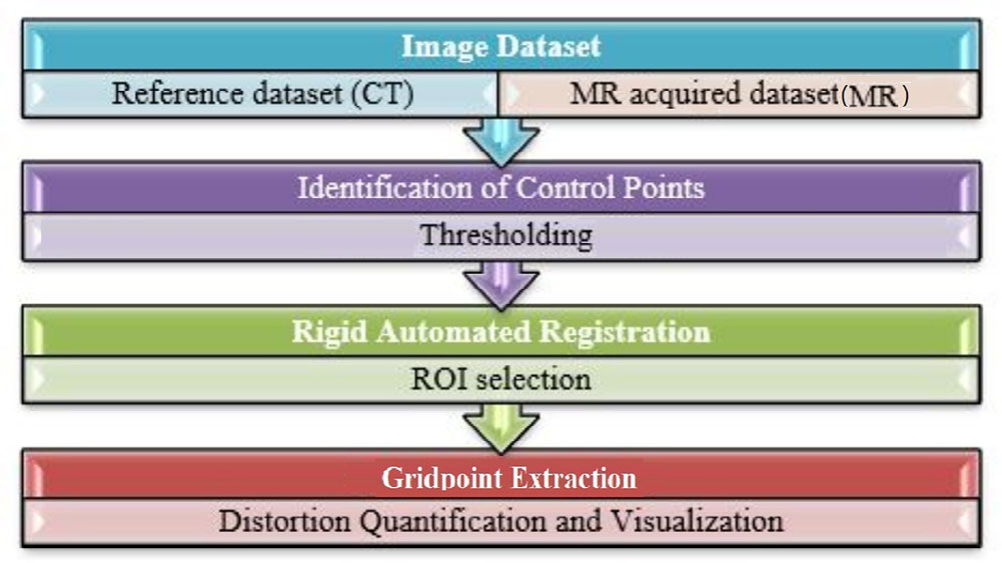
Flowchart of the distortion measurement method.

The user interface and backend processing pipeline of the software are shown in Figure 4. The grid‐point extraction methods follow the techniques proposed by Stanescu et al.^18^ After detecting all grid intersections, the software registers the MR control points to CT scan ground truth. Interpolation is then applied to generate the 3D distortion vector fields. To validate the software results, we scanned a real CT and compared the distortion data with reference data from the software to ensure accuracy. The images were also visually inspected for any artifacts and were found to be free of artifacts.

### 2.5 Distortion by outer radius

To evaluate the variation in maximum and mean total image distortion relative to the image volume, the outer radius of a spherical band was used as a reference. This approach involved analyzing distortions at increasing distances from the isocenter, ranging from 10 to 160 mm, within spherical bands. The maximum distortion represents the worst‐case error, while the mean distortion provides an average error within each band. By studying these distortions across different MRI sequences commonly used in the CK protocol, the analysis highlights how distortions increase with distance from the isocenter and vary depending on the sequence parameters. This helps in understanding and potentially correcting distortion patterns to improve imaging accuracy.

### 2.6 External beam planning

**FIGURE 5:**
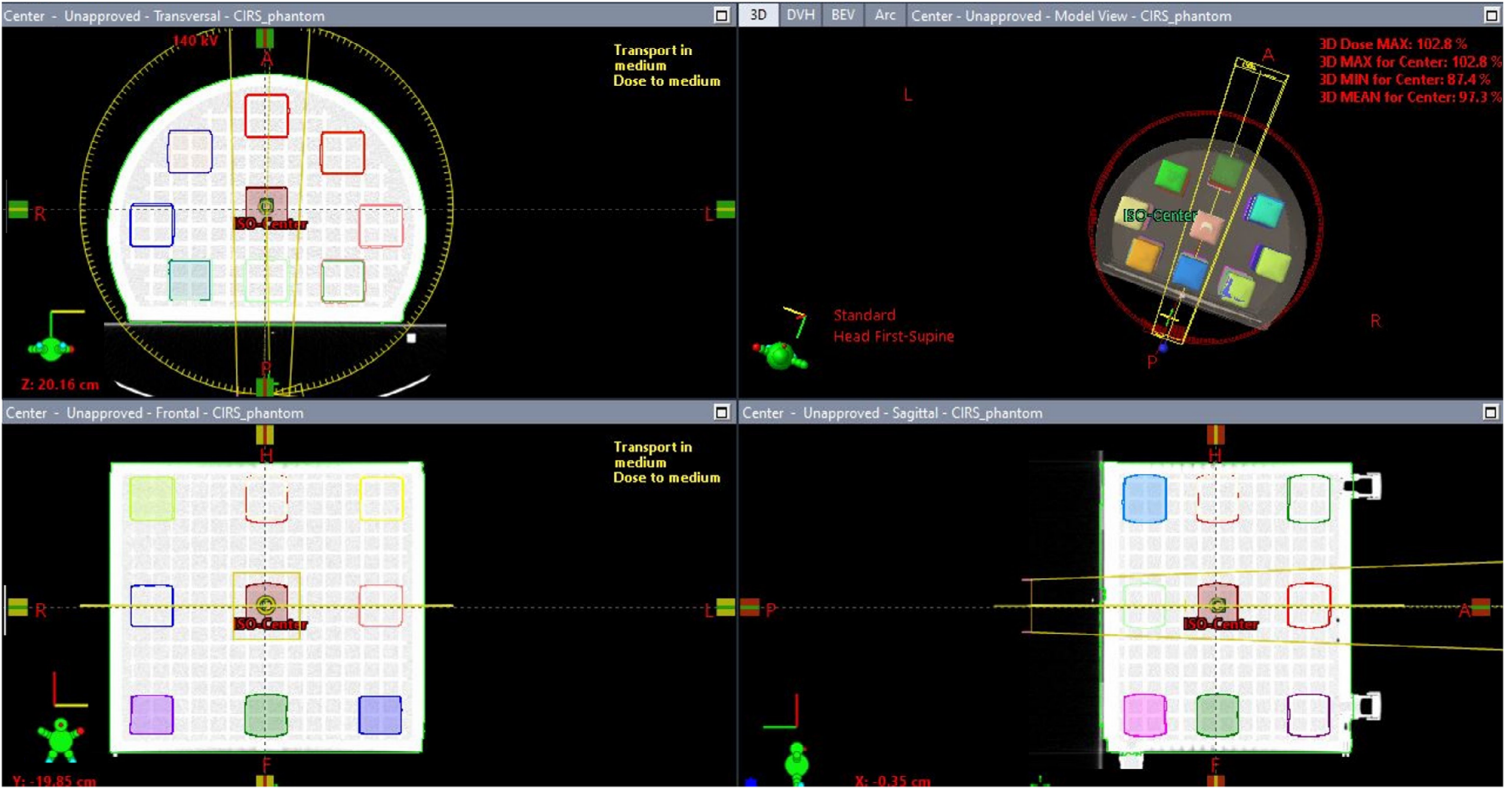
Mono isocentric coplanar single arc plan targeting central structure.

To assess the dosimetric impact of observed distortions, a highly conformal volumetric modulated arc therapy (VMAT) technique was used, utilizing a single full mono‐isocentric coplanar arc for each structure, as in Figure 5. Twenty‐seven MRI‐contoured structures were transferred to CT following rigid registration and incorporated into external beam treatment planning. VMAT plans using 6MV photon beams were generated in Eclipse V18.1 with the Acuros XB 18.1 dose calculation algorithm and a 1 mm dose grid resolution, prescribing 16 Gy in a single fraction to achieve 95% coverage of the GTV for all structures contoured in CT. The isocenter for treatment planning was placed at the center of each structure in CT. Delivered doses to each CT‐defined volume were compared with the corresponding MRI ‐transferred volumes to evaluate discrepancies.

### 2.7 Clinical dose evaluation

**FIGURE 6:**
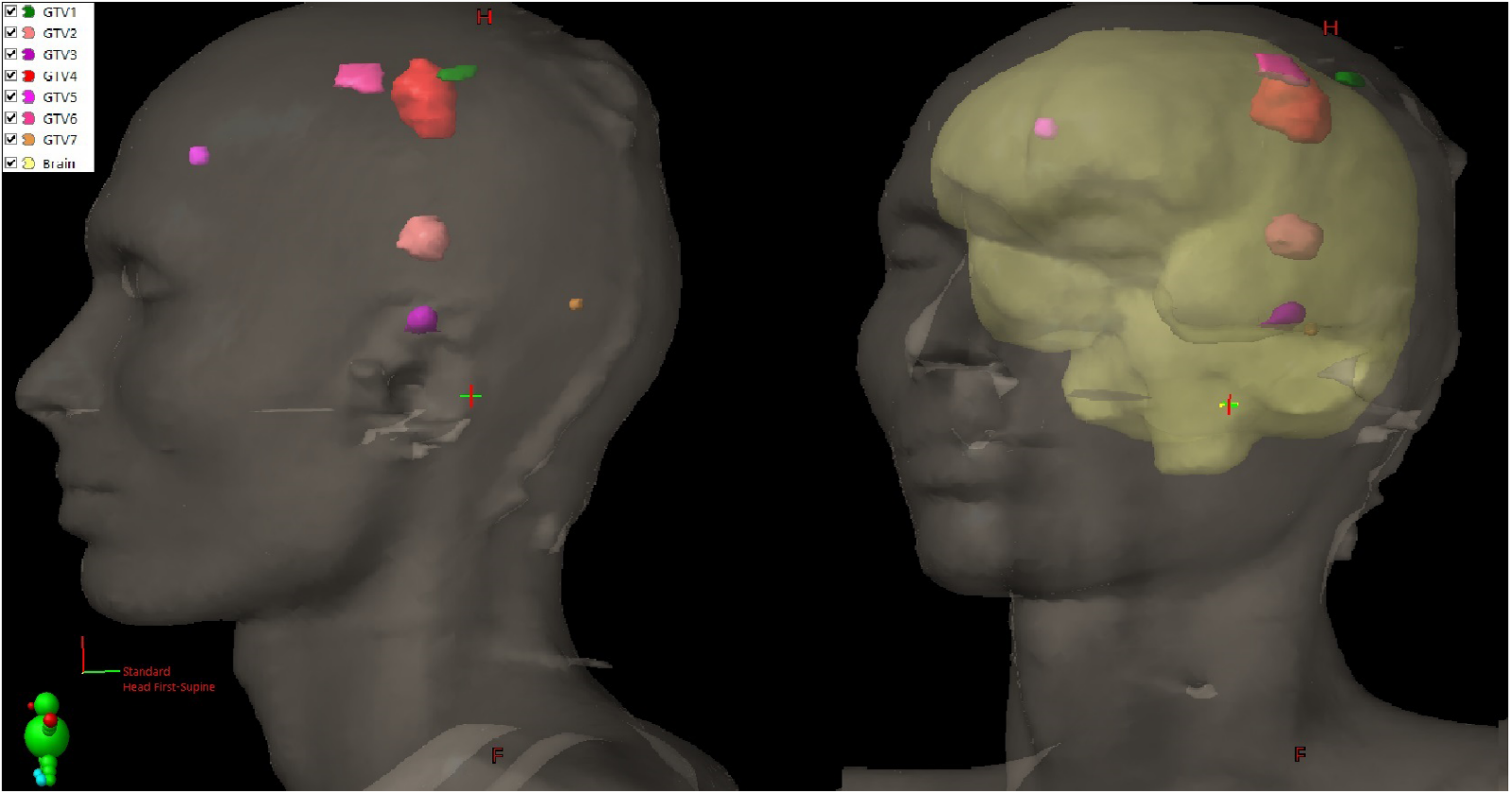
Lesions localization in lateral view (left) and oblique view (right) for better visualization of lesion shapes and locations.

A clinical SRS plan involving brain metastases with seven spatially scattered lesions (Figure 6) with diameters of 0.56, 0.72, 1.14, 1.76, 1.80, and 2.41 cm, respectively, was selected to correlate phantom‐based distortion measurements on patient‐specific data and to investigate the dosimetric impact of geometric distortion on it. A clinical single‐isocenter, multi‐target VMAT plan was selected, delivering 20 Gy in one fraction using 6FFF photon beams and four arcs. To simulate the impact of distortion on dose coverage, the isocenter of the original clinical plan was shifted by 3 mm, reflecting the maximum distortion measured in the MR CIRS phantom within the clinical field of view (FOV).

## 3 RESULTS

### 3.1 MR‐Sim QA

#### 3.1.1 B_0_ homogeneity

The data in Table 2 shows the mean B_0_ inhomogeneity measured in parts per million (ppm) over a quarterly period using the bandwidth‐difference option in the X, Y, and Z directions for a 27‐cm distance‐specific variation (DSV:27 cm). In the Z direction, homogeneity remained constant with an average of 0.11 ppm, while the X direction exhibited greater variability at 0.34 ppm. The Y direction showed moderate fluctuation, averaging 0.14 ppm. Overall, the data indicate a generally stable B_0_ performance, which helps minimize nonlinear distortions, particularly near the isocenter, where B_0_ is most homogenous.

**TABLE 2:** Mean quarterly B_0_ (main magnetic field) homogeneity through bandwidth difference option in x, y, and z directions.

DSV_27 cm_		Z direction (ppm)	X direction (ppm)	Y direction (ppm)
Bandwidth‐difference option	1st monthly	0.07	0.35	0.14
	2nd monthly	0.07	0.42	0.20
	3rd monthly	0.19	0.26	0.07
	**Average**	**0.11**	**0.34**	**0.14**

*Note*: B_0_ Homogeneity unit: parts per million (ppm) in a distance‐specific variation (DSV).

#### 3.1.2 ACR geometrical accuracy

**FIGURE 7:**
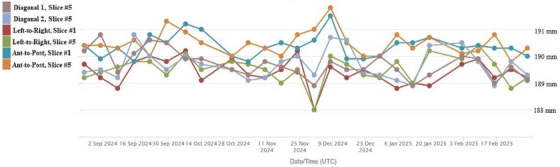
ACR MRI‐T1 axial geometrical accuracy assessed during weekly QA of 1.5T MR‐Sim. Results are for phantom slice #5 with an expected value of 190 ± 2 mm.

The MR‐Sim weekly QA contour plots and trend data confirm that the MRI system meets ACR recommendations for geometric accuracy, with distortions consistently below the 2 mm threshold.^17^ The deviation values include a maximum of 191.8 mm, a minimum of 188.0 mm, and a mean of 189.9 mm compared to the expected reference of 190 mm. A standard deviation of 0.64 mm was observed across all planes over 26 weeks of QA measurments (Figure 7).

### 3.2 MR geometrical evaluation

#### 3.2.1 MR geometrical correction assessment

**FIGURE 8:**
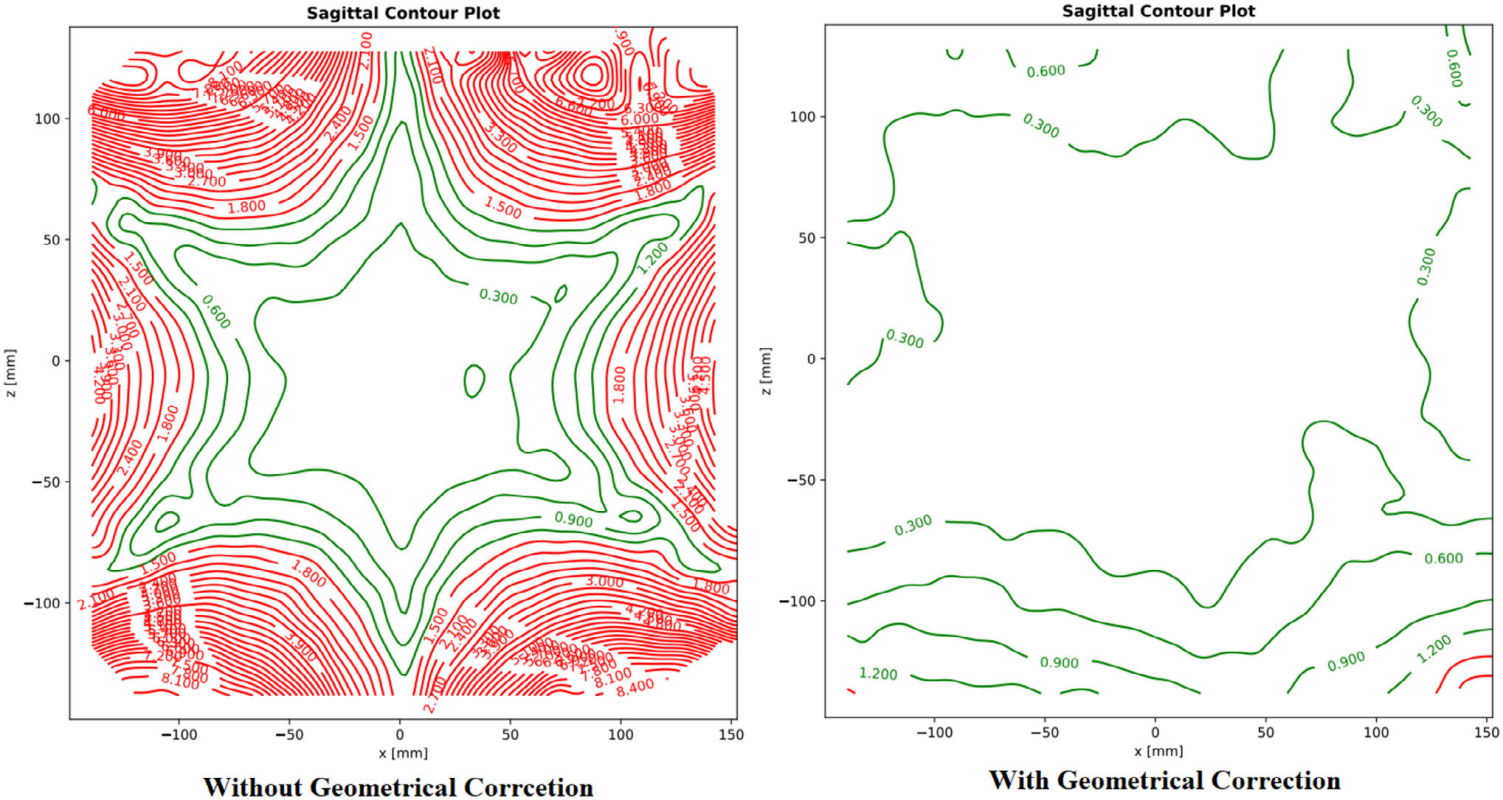
2D plots of MR geometric distortions in the sagittal plane with (right) and without (left) including geometric correction. Red contours indicate distortions > 1.5 mm tolerance. Enabling geometric correction (right) keeps distortion within acceptable limits across most of the plot.

Figure 8 demonstrates the impact of 3D geometric correction on a sagittal plane with a contoured plot of a T1 MPRAGE 3D sequence, both with and without geometric correction. The contour plots highlight the effectiveness of geometric correction in reducing image distortions. Without correction, the maximum distortion reaches approximately 8.4 mm within 30 cm FOV, particularly at the periphery, whereas with correction, it is reduced to around 1.2 mm—an 86% improvement. The mean distortion also decreases significantly, from over 1.4 mm to approximately 0.5 mm, indicating a 71% enhancement in geometric accuracy. Furthermore, the corrected plot exhibits more uniform contour line spacing, reflecting improved spatial consistency compared to the uncorrected plot, where the lines are irregular and distorted. These findings underscore the substantial benefits of applying geometric correction in minimizing distortions, especially in the peripheral regions.

#### 3.2.2 Absolute distortion in 3D and 2D MR sequences

**FIGURE 9:**
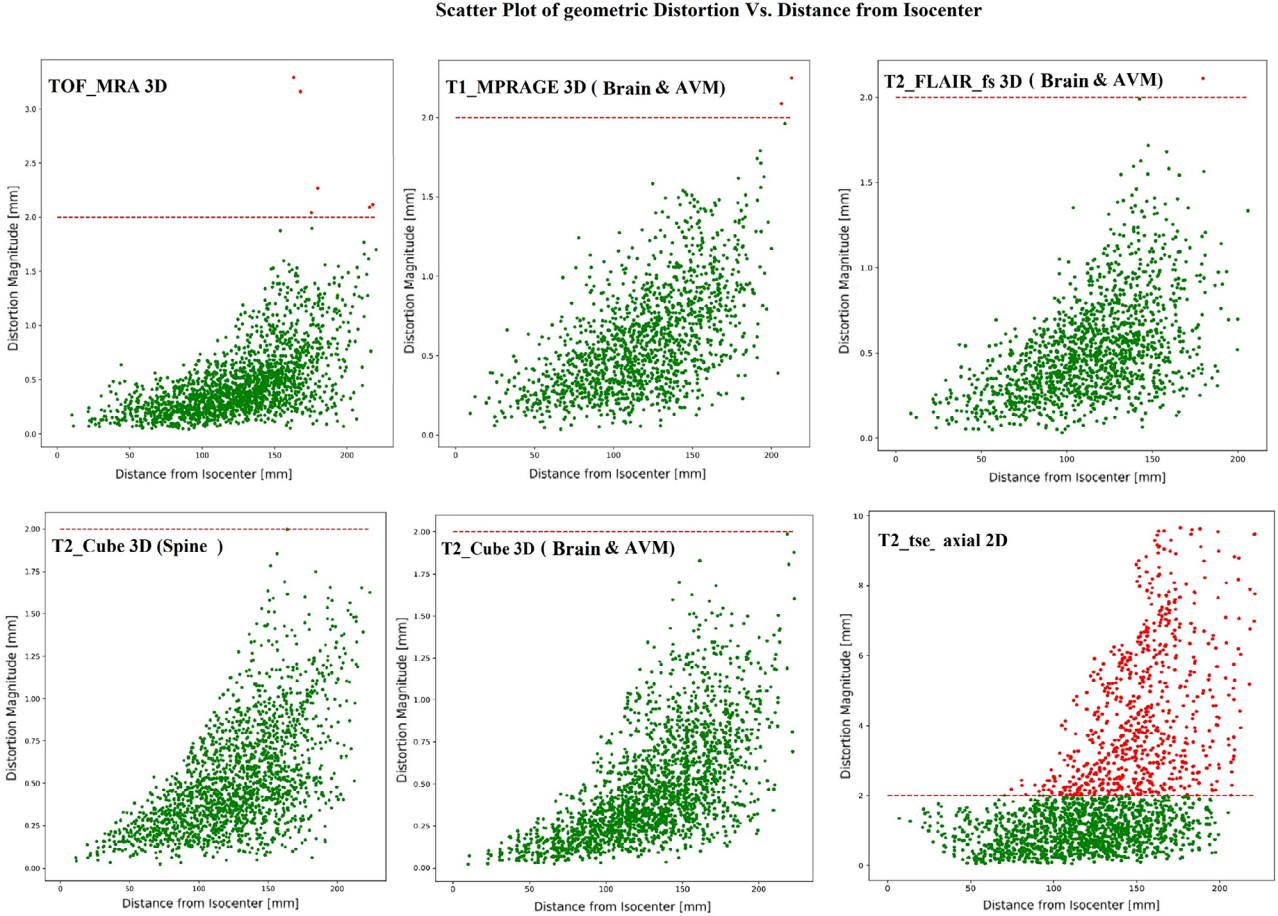
Scatter plots of geometric distortion (magnitude) are shown for all 3D MRI sequences used in SRS treatment (for brain, spine, and AVM using CK machine) and for a 2D MRI sequence without correction Horizontal lines indicate TG‐284 recommendation of 2 mm distortion tolerance in SRS.

The scatter plots in Figure 9 highlight the relationship between geometric distortion and distance from the isocenter across different clinical CK MRI sequences. Statistically, 3D sequences such as T1 MPRAGE, T2 FLAIR, and T2 Cube exhibit relatively low geometric distortion, with most data points concentrated below 1.5 mm and minimal outliers exceeding 2.0 mm. For instance, T1 MPRAGE 3D maintains distortions well below 1.5 mm, with only a few extreme outliers near 2.0 mm, while T2 Cube sequences demonstrate consistent performance with distortions rarely exceeding 1.75 mm. Additionally, TOF_MRA showes reliable results within 150 mm from the isocenter but exhibites higher distortions, with a maximum of 3.3 mm, in the peripheral regions of larger FOVs. Generally, no significant differences (*p* > 0.05) in distortion levels were observed among all 3D sequences, with maximum distortions consistently around 2.0 mm. However, a significant difference was observed in the 2D T2_TSE_Axial sequence (*p* < 0.001). The 2D sequence demonstrates significant limitations, with distortions approaching 10 mm for distances more than 100 mm from the isocenter. The 2D plot also displays a clear upward trend, with a large cluster of points between 2 and 10 mm, indicating poor geometric stability. Overall, 3D sequences provide statistically superior spatial accuracy, exhibiting smaller distortion ranges and fewer extreme values compared to 2D sequences, which show higher variability and larger distortion magnitudes in peripheral regions due to the lack of a geometrical correction option with current parameters.

#### 3.2.3 Cumulative MR sequence distortion (min., mean, and max.)

**FIGURE 10:**
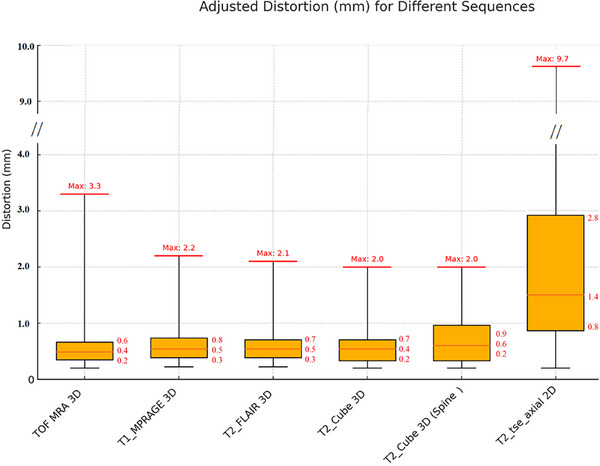
Boxplot showing distortion values for 3D T2 Cube/T2 FLAIR/T1 MPRAGE/3D TOF Angiography and 2D T2 MRI sequences for the CIRS MR distortion phantom. The orange horizontal line in the middle of each box shows the median value (*n* = 3 for each sequence).

The distortion analysis without considering the FOVs of different MRI sequences, presented in Figure 10, provides insights into the performance of each sequence in a geometrical phantom. All MRI sequences summarized in Table 1 were evaluated, and each was repeated three times (*n* = 3) to ensure statistical reliability.

As shown, the median distortion levels for each sequence are marked. Among the 3D sequences, both 3D T2 Cube (used in CK for AVM or other lesions) and 3D T2 FLAIR demonstrated low distortion levels. In contrast, the 3D TOF MRA sequence had the highest maximum distortion among the 3D sequences (3.3 mm), indicating a potential limitation in peripheral vessel delineation. However, the mean distortion for this sequence (0.4 mm) is within the tolerance recommended by AAPM TG‐284.^15^ Notably, the 2D T2 Axial sequence without 3D geometric correction exhibited the highest overall distortion, with a maximum value of 9.7 mm and significant variability and a mean value of 1.4 mm, reflecting its susceptibility to greater distortion.

#### 3.2.4 Mean and max. distortion by outer radius

**FIGURE 11:**
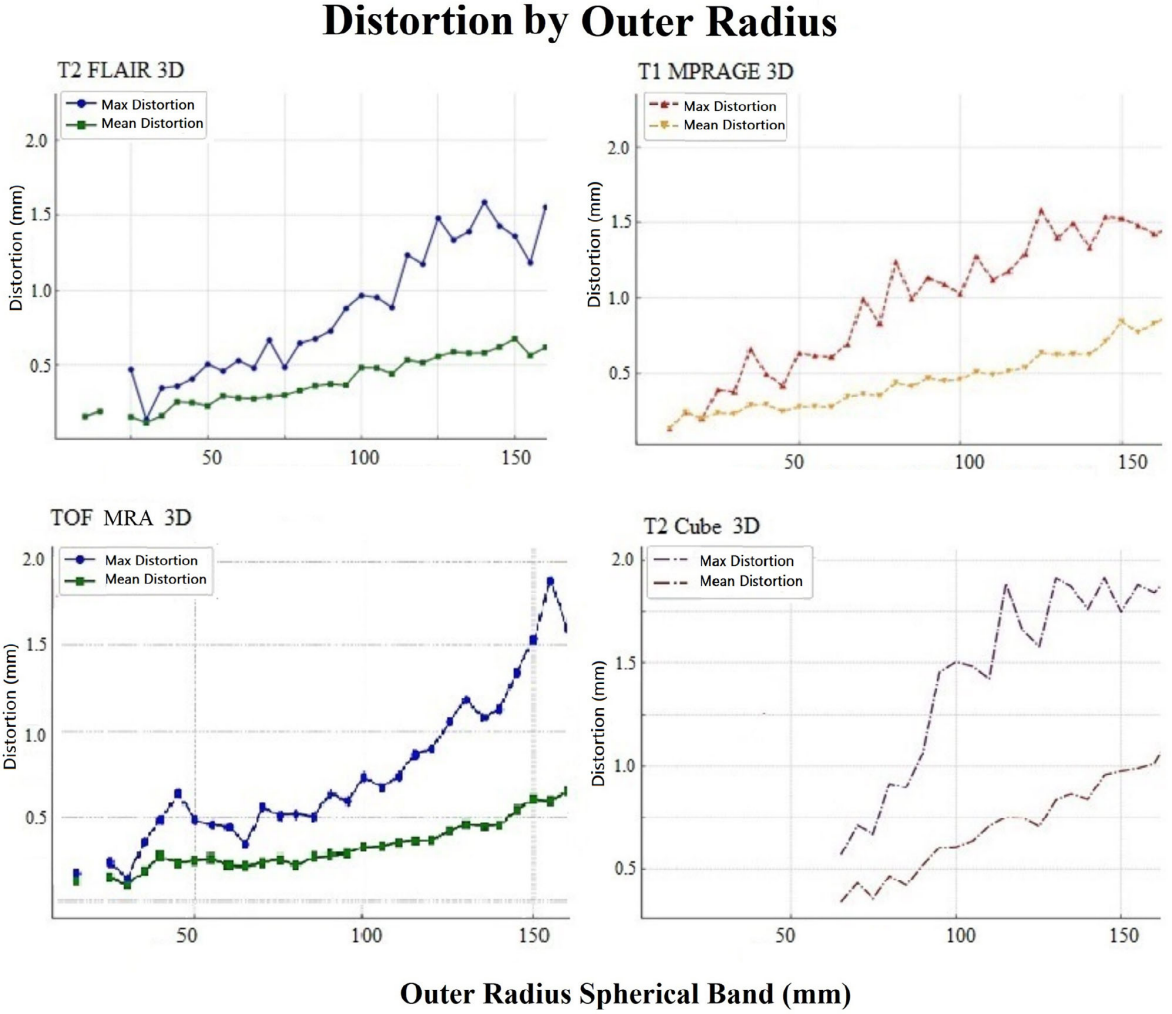
Maximum and mean magnitude of distortions (mm) over the outer radius spherical band in different MR sequences.

Evaluating max and mean distortion by outer radius reveals overall accuracy (mean) and worst‐case scenarios (max), highlighting spatial performance trends (Figure 11). This helps identify limitations in peripheral regions and guide improvements for reliable CK imaging. This analysis examines the relationship between error magnitude and outer radius across four imaging sequences: T2 FLAIR 3D, T1 MPRAGE 3D, TOF MRA 3D, and T2 Cube 3D. Across all sequences in Figure 11, both maximum and average distortion increase as the outer radius expands, reflecting a consistent decline in accuracy at greater distances from the center. Although T2 FLAIR 3D and T1 MPRAGE 3D show the most linear and steady growth in distortion, indicating robustness and fewer extreme outliers, T2 FLAIR 3D demonstrates moderate increases in average error but significant variability in maximum error with larger radii. In contrast, TOF MRA 3D and T2 Cube 3D exhibit more pronounced fluctuations and peaks in maximum distortion, with TOF MRA 3D reaching the highest error values near the outermost radii. These trends suggest that while T1 MPRAGE 3D and T2 FLAIR 3D might be more reliable for applications requiring high accuracy across a wide range of radii, TOF MRA 3D and T2 Cube 3D may need optimization to mitigate error spikes at greater distances.

### 3.3 Dosimetric impact

#### 3.3.1 Contour variability on rigid registration

Given the critical importance of accurate contouring and registration in dose distribution, the evaluation results obtained using Eclipse showed target registration errors with a mean of 0.1 mm and a standard deviation of 0.5 mm.

**FIGURE 12:**
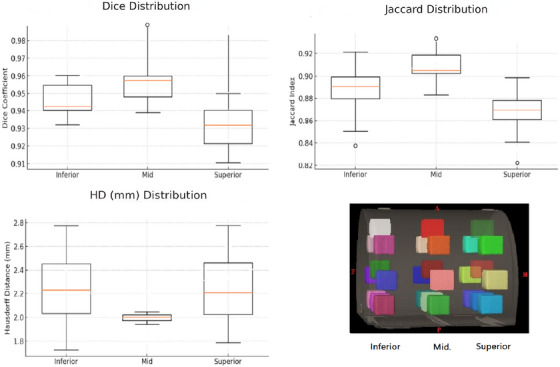
The box plot shows the average values of HD, Dice, and Jaccard indices for contour comparisons in each region across various contours in the CIRS phantom.

The contour evaluation, focusing on a clinical MR sequence, demonstrated a high degree of similarity across various regions of the phantom. The Dice and Jaccard indices showed mean values of 0.94 and 0.89, respectively, indicating significant contour overlap. Further analysis using the HD metric confirmed the findings, with average values of 2.1 mm, showing minimal deviation between CT and MR contours. This was particularly evident at the isocenter, which achieved a Jaccard value of 0.99. Notably, contour accuracy was higher in the central section of the phantom compared to the superior and inferior regions (Figure 12).

#### 3.3.2 Dosimetric accuracy across central and peripheral regions

The dosimetric analysis reveals significant variations in dose distribution across the phantom, with notable discrepancies between MR and CT imaging modalities (*p*‐value < 0.001 using both paired *t*‐test), as well as between central and peripheral structures. These differences highlight the impact of geometric distortions on treatment accuracy, particularly in the peripheral regions (Table 3).

**TABLE 3:** Mean dose and D_95%_ comparison of CT and MR contours across CIRS phantom using 27 VMAT plans.

No.	Geometrical index		D_95%_ [%] GTV				D_mean_ [cGy]		Volume [cm^3^]	
	Contour pairs	Jaccard	CT	MR	MR_Nor._	Diff.	CT	MR	CT	MR
1	Isocenter	0.99	96.6	96.3	95.7	0.9	1601.6	1600.3	99.0	99.6
2	Mid_Left	0.92	97.3	95.8	91.5	5.8	1606.5	1599.7	98.7	103.3
3	Mid_Right	0.92	95.1	92.8	86.7	8.4	1564.5	1556.3	96.4	103.2
4	Mid_Anterior	0.89	96.0	94.3	87.7	8.3	1580.3	1573.6	96.8	104.1
5	Mid_Posterior	0.91	96.8	93.5	87.2	9.6	1601.2	1589.5	96.6	103.6
4	Mid_Lt_Anterior	0.90	95.9	94.7	87.9	8.0	1581.0	1575.7	97.1	104.6
7	Mid_Lt_Posterior	0.88	95.7	93.6	87.4	8.3	1583.4	1574.7	96.3	103.1
8	Mid_Rt_Anterior	0.90	95.0	92.6	88.4	6.6	1566.6	1557.0	96.5	101.1
9	Mid_Rt_Posterior	0.90	96.0	93.0	87.7	8.3	1583.9	1572.4	96.5	102.3
10	Sup_Center (Head)	0.90	94.9	98.2	99.3	4.4	1578.6	1572.1	100.3	99.2
11	Sup_Left	0.88	94.7	93.1	86.8	7.9	1570.1	1566.0	104.6	112.2
12	Sup_Right	0.88	95.1	93.7	87.8	7.3	1566.9	1561.5	105.9	113.0
13	Sup_Anterior	0.88	96.0	93.8	85.6	10.4	1589.4	1581.0	105.8	115.9
14	Sup_Posterior	0.87	95.1	93.2	85.0	10.1	1572.4	1566.0	103.5	113.5
15	Sup_Lt_Anterior	0.88	96.8	95.0	86.2	10.6	1601.2	1593.5	104.9	115.6
16	Sup_Lt_Posterior	0.82	95.3	91.9	81.6	13.7	1568.9	1557.7	103.3	116.4
17	Sup_Rt_Anterior	0.86	94.4	91.5	83.1	11.3	1564.5	1553.2	102.3	112.7
18	Sup_Rt_Posterior	0.86	95.4	93.6	85.3	10.1	1570.3	1561.3	101.8	111.7
19	Inf_Center (Feet)	0.93	95.1	95.3	96.5	1.4	1577.3	1578.1	98.3	97.1
20	Inf_Left	0.90	94.5	91.5	83.2	11.3	1575.3	1571.8	99.8	109.7
21	Inf_Right	0.87	96.3	95.1	85.1	11.2	1585.8	1580.5	98.7	110.3
22	Inf_Anterior	0.90	94.4	93.2	85.1	9.3	1554.4	1546.2	105.8	115.9
23	Inf_Posterior	0.88	95.1	94.0	87.6	7.5	1577.3	1572.3	96.6	103.6
24	Inf_Lt_Anterior	0.90	92.7	90.7	82.6	10.1	1575.3	1571.8	97.4	107.0
25	Inf_Lt_Posterior	0.88	93.7	91.3	83.5	10.2	1566.2	1557.8	98.2	107.4
26	Inf_Rt_Anterior	0.89	93.8	90.9	83.1	10.7	1562.8	1553.8	98.8	108.1
27	Inf_Rt_Posterior	0.85	95.6	94.0	84.0	11.6	1583.3	1578.6	97.9	109.5

Isocenter structure (No. 1) exhibits the most stable dosimetric performance, with D_95%_ values of 96.6% (CT) and 95.7% (MR), showing a minimal 0.9% in the MR_normalized_ difference. (MR_normalized_: MR D_95%_ is normalized to the same structure's volume in CT). Its D_mean_ values (1601.6 vs. 1600.3 cGy) and Jaccard index (0.99) indicate minimal geometric distortion and high dose conformity.

Among central structures (No. 1, 10, and 19), the isocenter maintains the highest stability, whereas the superior and inferior centers show relative D95% differences of 4.4% and 1.4%, respectively. These deviations, though small, highlight the need for careful consideration in treatment planning to ensure optimal dose coverage.

Peripheral structures show greater dosimetric variability than central ones, with MR_normalized_ D_95%_ values of 91.5%, 83.2%, and 85.1% in the middle, inferior, and superior sections, respectively. Their deviations from central reference values (5.8%, 11.3%, and 10.4%) indicate reduced dose conformity. Peripheral structures in the middle section exhibit the least deviation, suggesting better stability. A decreasing Jaccard index further reflects reduced dose coverage, potentially compromising treatment precision and dose reliability. Refer to Section 2.3 for the location of structures.

#### 3.3.3 DVH comparison of central structures

**FIGURE 13:**
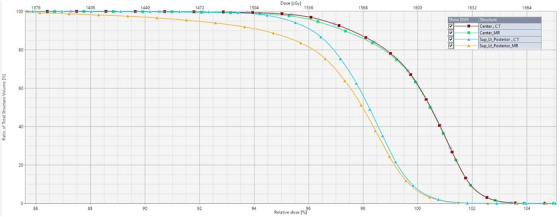
DVH comparison between MR and CT‐based treatment plans in central and most deviated structures.

Figure 13 presents a dose‐volume histogram (DVH) comparing CT and MR dose distributions for a central structure (isocenter) and one peripheral structure (Sup_Lt_Posterior with the greatest geometric error). The CT DVH serves as the reference for each pair.

For the central structure, the MR DVH closely aligns with the CT reference, indicating minimal geometric distortion and consistent dose coverage (D_95%_: 96.3% MR vs. 96.6% CT). The mean dose (D_mean_) for MR (1600.3 cGy) also closely matches CT (1601.6 cGy), confirming accuracy near the isocenter.

Conversely, the MR DVH for the peripheral Sup_Lt_Posterior structure exhibits a broader slope, indicating dose inhomogeneity and decline (D_95%_: 91.9% MR vs. 95.3% CT), with a notable dose difference of 13.7%. The D_mean_ for the peripheral structure is also reduced (1557.7 cGy MR vs. 1568.9 cGy CT), while the MR structure's volume expands by ∼12.7% relative to CT, indicating distortion‐related volumetric changes.

These findings emphasize the importance of evaluating peripheral dose distributions, as increased geometric distortion at greater distances from the isocenter can affect treatment accuracy, necessitating adjustments to mitigate potential dosimetric discrepancies.

#### 3.3.4 Clinical dose evaluation

Compared to the original plan, a 3 mm plan shift led to reduce all GTVs dose coverage. GTV7 was most affected, with coverage falling from 95.2% to 89.7%, failing to meet the clinical target in all plans. GTVs 1, 2, and 5 showed moderate sensitivity, with some plans dropping below the ≥98% coverage threshold. Although GTVs 3 and 4 maintained coverage above 98%, they still experienced slight decreases (Table 4).

**TABLE 4:** Dose coverage comparison of lesions in original and shifted plans.

	Contours						
	GTV1^a^	GTV2	GTV3	GTV4	GTV5	GTV6	GTV7
Clinical goal (cGy) ≥ 98%	V_1800_	V_2000_	V_2000_	V_1800_	V_2000_	V_2000_	V_2000_
Original plan coverage (%)	98.6	99.4	99.2	99.2	97.6	99.0	95.2
Shifted plan coverage (%)	96.8	97.6	98.8	98.7	95.5	97.8	89.7

^a^ Skull bone lesion.

## 4 DISCUSSION

Geometric distortion in MRI can compromise the spatial accuracy essential for RT planning. This study extends prior work (6, 9, 11, 16) by directly linking phantom‐measured distortions to dosimetric consequences in high‐precision treatments. Using the CIRS Model 604‐GS phantom, we systematically quantified distortions across clinical 2D and 3D sequences at 1.5 T MR‐Sim.

Our results confirm that 2D T2 TSE Axial sequence exhibits up to four‐fold greater mean distortion (≈1.4 mm) and a maximum error of 9.7 mm at 150 mm off‐isocenter, underscoring their vulnerability when gradient correction is limited. In contrast, 3D volumetric sequences (T1 MPRAGE, T2 FLAIR, T2 Cube) maintained submillimeter average distortions (≈0.4 mm) and a maximum of 2.2 mm, in line with AAPM TG‐284 recommendations.^4^, ^18^

Importantly, distortion magnitude increased with radial distance, reaching 3.3 mm in TOF MRA at the phantom periphery—a finding particularly relevant for CyberKnife treatments targeting off‐axis lesions. Phantom‐derived shifts (up to 3 mm) applied to a clinical SRS VMAT plan resulted in average GTV coverage losses of 1.9%, with lesions (e.g., max. 5.5% for GTV7) failing to meet the ≥98% threshold under combined XYZ (3 mm) distortion.

Dosimetric discrepancies between MRI‐ and CT‐based planning were minimal (<1% in central regions) but escalated to 13.7% in peripheral structures, highlighting that peripheral targets are at greatest risk of underdosage if distortions are uncorrected.

These findings demonstrate that sequence selection and geometric correction algorithms are crucial to mitigate distortion, particularly for off‐center targets in stereotactic applications. Limitations include scanner‐specific variability and the influence of patient anatomy on distortion patterns. Future work should refine distortion‐correction workflows, explore real‐time mapping techniques, and validate adaptive planning strategies that incorporate measured geometric errors.

## 5 CONCLUSION

This study evaluates the geometric accuracy of MRI protocols for RT planning using a 1.5T MR‐Sim scanner and a geometrical phantom. While 3D sequences show acceptable accuracy, significant variability was observed, with the current T2 TSE 2D sequence exhibiting higher distortion. 3D T1 MPRAGE, 3D T2 Cube, and 3D T2 FLAIR provide the best balance of low distortion and stability, while the 3D TOF MR Angiography sequence may require caution for peripheral delineations.

The results confirm that central anatomical structures align well in MRI‐CT registration, ensuring accurate dose distribution. However, peripheral regions are more sensitive to geometric misalignments, emphasizing the need for careful protocol selection and patient positioning based on lesion location. Optimized MRI techniques and image fusion strategies are also essential for precise treatment planning.

## AUTHOR CONTRIBUTIONS

Mojtaba Barzegar performed measurements and analysis, contributed to VMAT planning, co‐authored sections, created figures, and manuscript writing. Aram Rostami designed the study, directed all content, contributed to image registration and dosimetric evaluation sections, co‐authored sections, and proofread the manuscript. Abbas Yousef Mkanna contributed to VMAT planning, and dosimetric revisions. Satheesh Prasad Paloor revised the manuscript, and proofread all content. Ahamed Basith contributed to VMAT planning, Tarraf Torfeh and Souha Aouadi evaluated image distortion. Rabih Hamoud and Noora Al Hammadi provided all materials.

## CONFLICT OF INTEREST STATEMENT

The authors declare no conflicts of interest.
